# Concurrent Vibrio cholerae O1 Meningitis and Intracranial Lesions in a Patient With Sickle Cell Disease: A Case Report

**DOI:** 10.7759/cureus.53802

**Published:** 2024-02-07

**Authors:** Turki A Alshuaibi, Faisal A Althobaiti, Omar A Youldash, Riyadh O Shati, Bashayr Alamri

**Affiliations:** 1 Department of Hematology, King Fahad General Hospital, Jeddah, SAU; 2 Department of Neurology, King Fahad General Hospital, Jeddah, SAU; 3 Department of Internal Medicine, King Fahad General Hospital, Jeddah, SAU

**Keywords:** vibrio cholera, meningitis, sickle cell disease: scd, tuberculoma, vibrio cholerae o1 meningitis

## Abstract

Cholera meningitis is a rare complication of *Vibrio cholerae (V. cholerae)* infection. We present a case of cholera meningitis caused by toxigenic *V. cholerae* O1 in a 34-year-old male with sickle cell disease (SCD). The patient presented with fever, diarrhea, and altered mental status. Cerebrospinal fluid (CSF) analysis showed 5,231 cells/μL (53.9% neutrophils), a protein level of 462 mg/dL, and a glycorrhachia level of 26 mg/dL. *V. cholerae* O1 was isolated on CSF culture. Despite the patient undergoing antimicrobial therapy, brain imaging revealed basal ganglia ring-enhancing lesions suggestive of tuberculomas. Antituberculosis treatment and steroids led to clinical improvement. This report highlights the need to consider *V. cholerae* meningitis in patients with SCD who present with diarrhea and altered mental status. Prompt diagnosis and appropriate antimicrobial therapy are keys to improving patient outcomes.

## Introduction

Cholera is an acute diarrheal illness caused by toxigenic strains of the bacterium* Vibrio cholerae (V. cholerae)* serogroups O1 and O139. Although most cases are asymptomatic, symptomatic infections can result in copious watery diarrhea, leading to dehydration and even death if left untreated. Extraintestinal infections with *V. cholerae*, including bacteremia and meningitis, are rare but have been reported [1-9]. We present a rare case of *V. cholerae* O1 meningitis and intracranial tuberculomas occurring concurrently in a patient with sickle cell disease (SCD). This highly unusual dual central nervous system (CNS) infection highlights the increased susceptibility to unusual infections among patients with SCD, and the need for prompt neuroimaging and appropriate antimicrobial therapy. Early diagnosis and treatment are imperative to prevent neurological damage or death.

## Case presentation

A 34-year-old Saudi male with known SCD presented with a two-month history of persistent fever that had reached 40°C in the past two days and was accompanied by night sweats. Two days before the presentation, he had developed watery diarrhea with up to eight bowel movements per day. The stools were large in volume, grayish-yellow in color, and malodorous, without visible blood or mucus. A few hours after the presentation, he developed an altered mental status with disorientation and agitation. He had no history of seizures and no recent travel history. The patient did not have any cough, hemoptysis, or shortness of breath. He had no history of injection drug use and no known tuberculosis exposure.

The patient’s medical history included SCD, which had been treated with hydroxyurea and folic acid. Two years prior, he had developed diarrhea for two days, followed by behavioral changes, and had been hospitalized for 22 days for meningoencephalitis. The workup at that time included cerebrospinal fluid (CSF) analysis showing a white blood cell count of 13 cells/μL (46% monocytes), a protein level of 53 mg/dL, and a glycorrhachia level of 99 mg/dL (serum glucose: 110 mg/dL). The CSF, blood, and urine cultures had been negative. He had been treated with antibiotics at that time. In the subsequent two years, he had been relatively stable on hydroxyurea and folic acid with no major sickle cell crises.

On admission to our hospital, a physical examination revealed a temperature of 39.5 °C, a heart rate of 112 beats/minute, a respiratory rate of 18 breaths/minute, blood pressure of 134/86 mmHg, and oxygen saturation of 96% on room air. The patient was somnolent and disoriented about time, people, and place. He opened his eyes spontaneously and followed commands intermittently. He had no neck stiffness and Kernig's or Brudzinski's signs. His pupils were equal and reactive to light. His cranial nerves were intact. Motor examination revealed normal bulk and tone, with 5/5 strength throughout. Sensation was intact in all modalities. The reflexes were 2+ and symmetrical. Cerebellar testing was limited owing to his somnolence. The plantar reflex was upgoing on the right and downgoing on the left.

**Figure 1:**
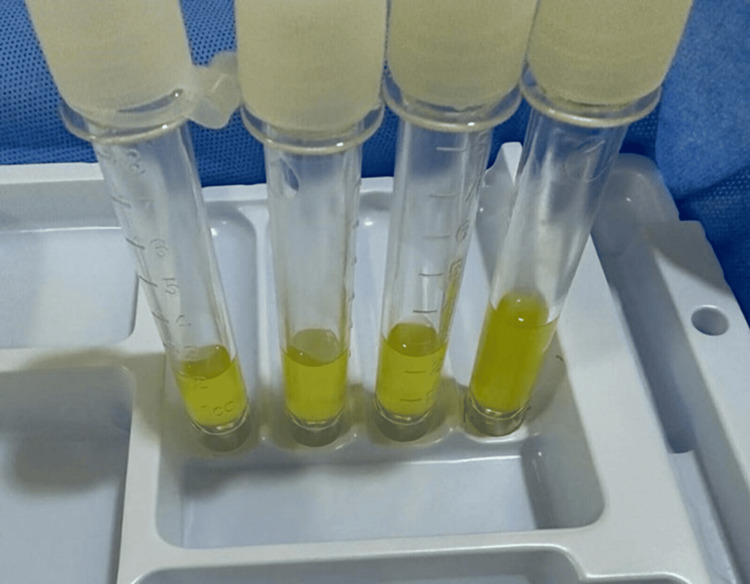
Initial lumbar puncture showing yellow turbid cerebrospinal fluid

Hematology revealed a white blood cell count of 12 × 10^9^/L and a neutrophil percentage of 78%. CSF analysis revealed yellow turbid fluid (Figure 1) with 5,231 cells/µL (53.9% neutrophils and 43.4% monocytes), a protein level of 462 mg/dL, and glycorrhachia level of 26 mg/dL (serum glucose: 139 mg/dL). CSF cytology revealed an inflammatory infiltrate with neutrophil predominance and occasional macrophages. The initial Gram staining was negative. *V. cholerae* was cultured from the CSF and blood. The serotype test results were positive for O1 and negative for O139. CSF analysis revealed negative results for acid-fast bacilli on smear microscopy and *Mycobacterium tuberculosis (M. tuberculosis)* on polymerase chain reaction (PCR) testing. The CSF culture for mycobacteria was also negative. Stool cultures showed no pathogenic bacterial growth, and urine cultures showed no bacterial growth. Serological testing was negative for HIV, hepatitis B virus, hepatitis C virus, and *Toxoplasma gondii*. Brain CT and chest radiography were unremarkable.

**Figure 2:**
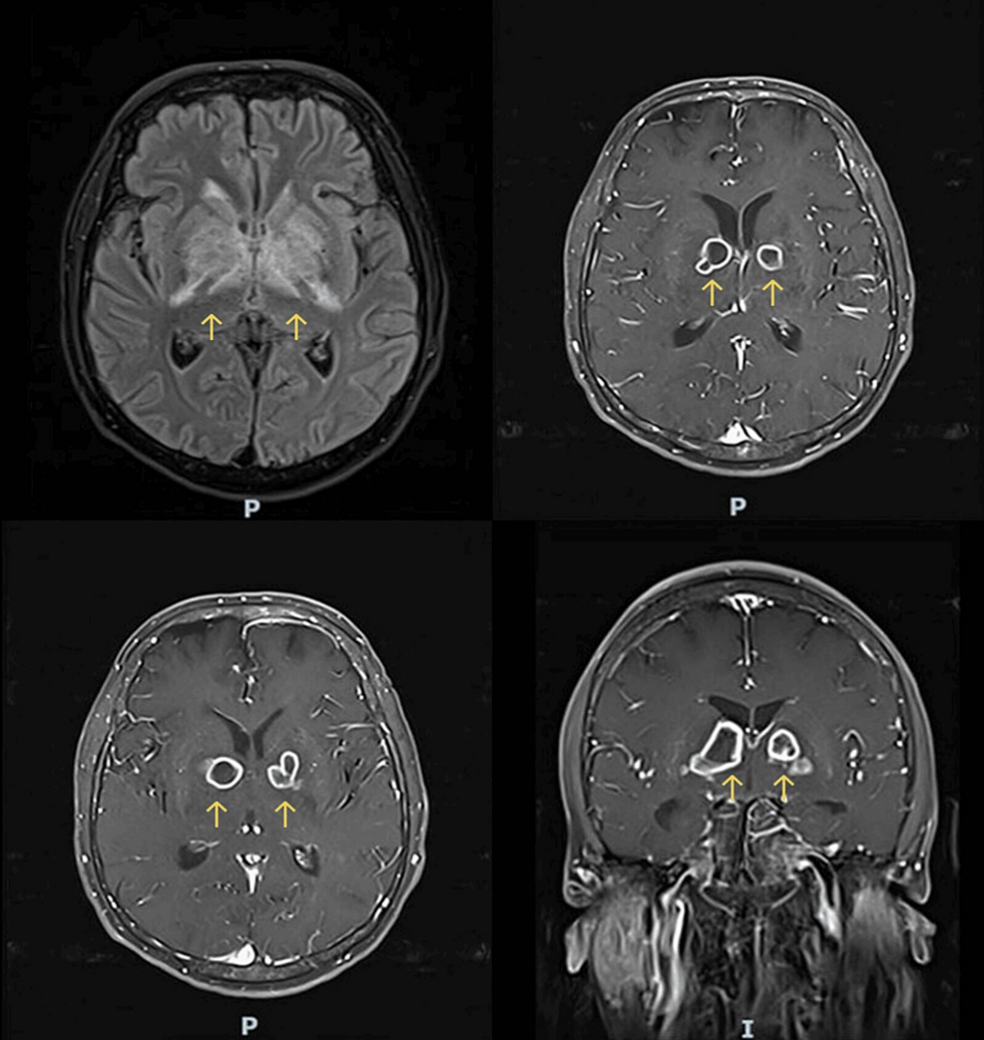
Initial brain MRI showing ring-enhancing lesion affecting both basal ganglia with surrounding edema MRI: magnetic resonance imaging

**Figure 3:**
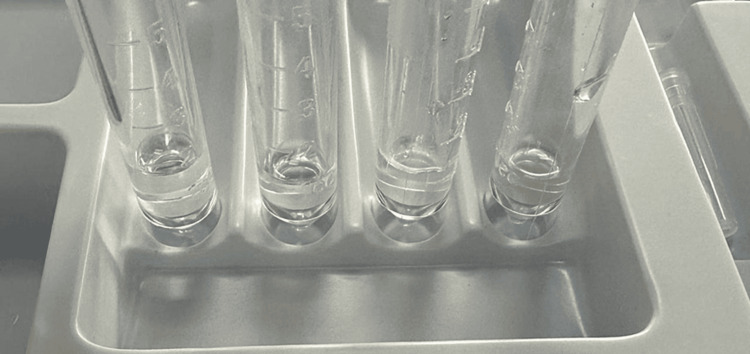
Repeat lumbar puncture showing clear cerebrospinal fluid

The patient was started on empiric intravenous ceftriaxone (2 g every 12 hours), vancomycin (1 g every 12 hours), acyclovir (700 mg every eight hours), and dexamethasone (10 mg every six hours). After two days, the CSF culture returned positive for *V. cholerae* O1. At this point, antimicrobial therapy was limited to intravenous ceftriaxone (2 g every 12 hours) and intravenous doxycycline (100 mg every 12 hours), and dexamethasone and acyclovir were discontinued. Contrast-enhanced MRI revealed bilateral ring enhancement lesions in the basal ganglia (Figure 2). The patient’s condition did not improve after two weeks of antimicrobial therapy. Repeated lumbar punctures showed clear CSF (Figure 3) with 5 cells/µL (60% lymphocytes), a protein level of 85 mg/dL, and a glycorrhachia level of 56 mg/dL (serum glucose: 130 mg/dL). CSF Gram staining was negative and CSF cultures showed no growth, including no *M. tuberculosis* on mycobacterial culture. *M. tuberculosis* PCR of the CSF was also negative.

**Figure 4:**
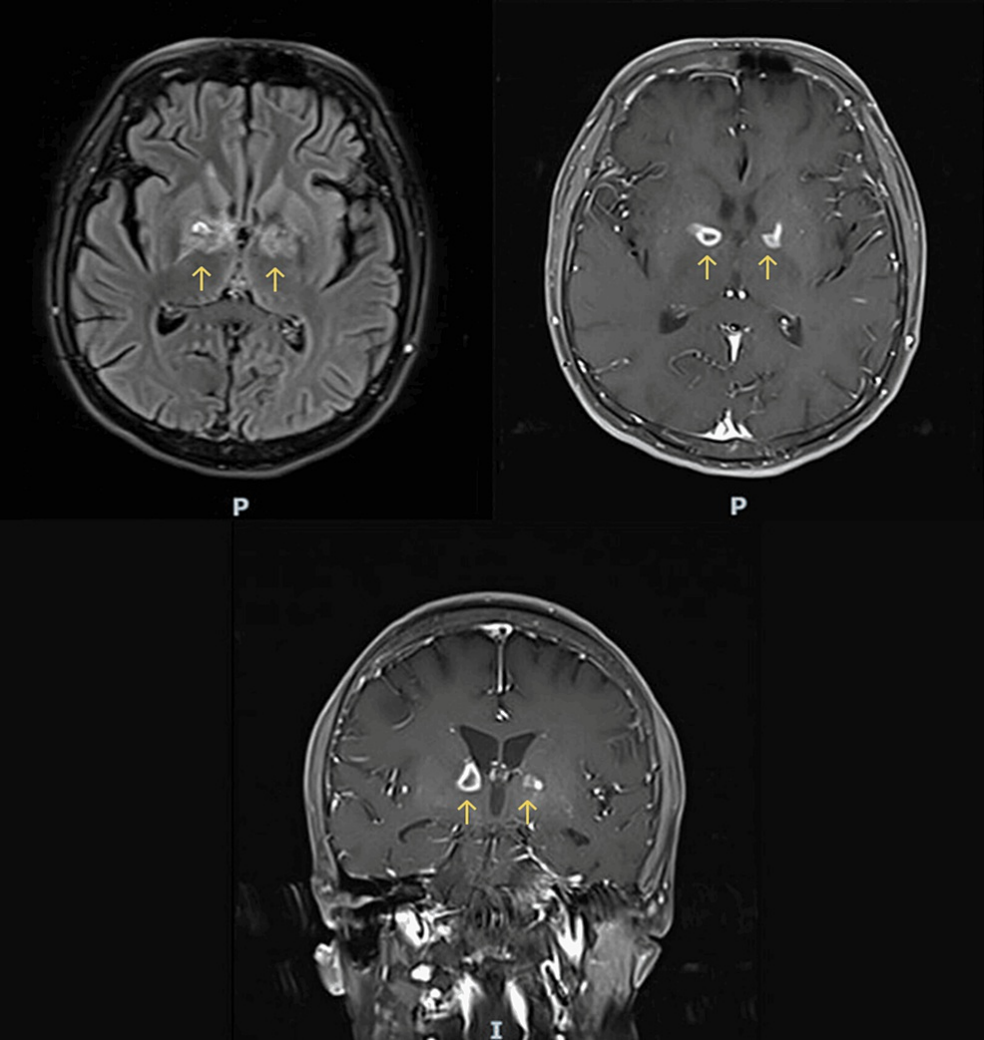
Repeat brain MRI showing significant improvement with marked regression of bilateral basal ganglia ring-enhancing lesions as well as reduction of edema MRI: magnetic resonance imaging

Despite the negative laboratory findings including negative CSF acid-fast bacilli smears, cultures, and PCR, a presumptive clinical diagnosis of tuberculomas was made based on the ring-enhancing lesions on MRI, elevated CSF protein, lack of improvement following appropriate antibiotic therapy for *V. cholerae* meningitis, and the patient's residence in an region endemic for tuberculosis. After a multidisciplinary evaluation, the patient was started on weight-based antituberculous therapy with isoniazid, rifampin, ethambutol, and pyrazinamide. Dexamethasone had been discontinued when the antibiotics were de-escalated. Dexamethasone was subsequently reinitiated along with antituberculosis therapy. Three weeks after starting antituberculous therapy, the patient showed a marked clinical improvement. On examination, he was attentive, alert, and oriented for people, time, and place. Repeated brain MRI showed a significant decrease in edema and shrinking of the ring enhancement lesions (Figure 4). After four weeks of antituberculous therapy and dexamethasone, the patient was discharged from the hospital in a stable condition on rifampin, isoniazid, pyrazinamide, and ethambutol for a further 32 days, followed by rifampin and isoniazid for 11 months with the addition of pyridoxine. Oral dexamethasone was tapered over four weeks upon discharge. At the follow-up four weeks after discharge, he remained asymptomatic, and routine laboratory test results were unremarkable.

## Discussion

CNS infections constitute a rare complication of cholera. Only a few cases of cholera meningitis have been reported in the literature so far, mostly in infants and neonates [1-6], with very few cases described in adults [7-9]. Impaired splenic function and defects in complement activation probably contribute to an increased susceptibility to invasive bacterial diseases in SCD [10]. While the pathogenesis of cholera meningitis is not completely understood, it is hypothesized to involve transient *V. cholerae* bacteremia, enabling hematogenous spread to the meninges [11]. The isolation of toxigenic *V. cholerae* O1 from the CSF implies that this strain has enhanced virulence and invasive potential when compared with other strains.

The administration of antimicrobial therapy is crucial in the management of severe infections and septicemia caused by *V. cholerae*. The selection of an appropriate antibiotic should be based on local susceptibility data. Doxycycline is the primary choice for treating infections caused by *V. cholerae* O1 or O139 in adults. However, erythromycin or azithromycin are recommended as alternatives in children and pregnant women. In cases where the infection is caused by *V. cholerae* non-O1/non-O139 strains, the use of ciprofloxacin and/or third-generation cephalosporins such as ceftazidime and ceftriaxone is recommended [12,13]. An important aspect of this case was the fact that alternative diagnoses were considered when the patient's condition failed to improve following appropriate initial therapy. The basal ganglia lesions found on MRI were critical in raising suspicion of CNS tuberculosis. Early neuroimaging and initiation of antituberculous therapy were key to the successful outcome in our patient.

This report has a few limitations, especially the absence of a genetic analysis of the *V. cholerae* strain and the inability to trace the source of infection. The diagnosis of CNS tuberculosis was inferred from neuroimaging and the patient’s response to antituberculous therapy in the absence of a tissue biopsy or a positive CSF culture for *M. tuberculosis*. However, despite these limitations, we believe this case adds to the limited literature on cholera meningitis and underscores the need for the prompt diagnosis and treatment of dual CNS infections.

## Conclusions

This case report highlights the need to consider *V. cholerae* O1 in the differential diagnosis of meningitis in patients with SCD and concomitant diarrhea. Given the high morbidity and mortality associated with the condition, prompt diagnosis and appropriate antimicrobial therapy are essential to achieving optimal outcomes. The isolation of toxigenic *V. cholerae* from the CSF warrants a public health investigation to determine its source and limit further transmission. Further genetic analysis of the strain could uncover virulent and invasive factors. Also, studies involving long-term follow-ups are needed to characterize the neurological sequelae of cholera meningitis and assess the response to treatment.
